# Postbiotics and Their Potential Applications in Early Life Nutrition and Beyond

**DOI:** 10.3390/ijms20194673

**Published:** 2019-09-20

**Authors:** Carrie A. M. Wegh, Sharon Y. Geerlings, Jan Knol, Guus Roeselers, Clara Belzer

**Affiliations:** 1 Laboratory of Microbiology, Wageningen University and Research, 6708 WE Wageningen, The Netherlands; carrie.wegh@wur.nl (C.A.M.W.); sharon.geerlings@wur.nl (S.Y.G.); jan.knol@danone.com (J.K.); 2 Danone-Nutricia Research, 3584 CT Utrecht, The Netherlands; guus.roeselers@danone.com

**Keywords:** gut microbiota, fermented infant formula, postbiotics

## Abstract

Postbiotics are functional bioactive compounds, generated in a matrix during fermentation, which may be used to promote health. The term postbiotics can be regarded as an umbrella term for all synonyms and related terms of these microbial fermentation components. Therefore, postbiotics can include many different constituents including metabolites, short-chain fatty acids (SCFAs), microbial cell fractions, functional proteins, extracellular polysaccharides (EPS), cell lysates, teichoic acid, peptidoglycan-derived muropeptides and pili-type structures. Postbiotics is also a rather new term in the ‘-biotics’ field. Where consensus exists for the definitions of pre- and probiotics, this is not yet the case for postbiotics. Here we propose a working definition and review currently known postbiotic compounds, their proposed mechanisms, clinical evidence and potential applications. Research to date indicates that postbiotics can have direct immunomodulatory and clinically relevant effects and evidence can be found for the use of postbiotics in healthy individuals to improve overall health and to relief symptoms in a range of diseases such as infant colic and in adults atopic dermatitis and different causes of diarrhea.

## 1. Introduction 

The microbial habitat within the human gastro-intestinal tract is the site of a complex and dynamic mutualistic relationship between the gut microbiota and the host. With this mostly mutualistic relationship, the gut microbiota influence a multitude of physiological functions in the host, often mediated via the host’s immune system [1]. The members of the gut microbiota also produce a wide range of compounds that can be used by both the host and by other microorganisms. Such relations are referred to as the host-microbe and microbial community interactions [2], and will be discussed later in this review. These interactions are vital for shaping the host microbial symbiosis and the establishment of stable communities that are health promoting and resilient to perturbation throughout life [3,4].

Although more research is required to determine the exact roles of the gut microbiota in specific aspects of health and disease, the microbiota composition is strongly correlated with a range of diseases and has become an important target for therapy and nutrition [5,6]. Gut microbiota composition and function can be modulated in several ways. The term ‘-biotics’ refers to nutritional strategies that can be utilized to direct the gut microbiota towards a more favorable state for host health. The term ‘biotic’ is derived from the Greek word biōtikós, meaning ‘pertaining to life’, and refers to the biological ecosystem made up of living organisms together with their physical environment [7]. Prebiotics, probiotics and synbiotics can modulate the gut microbiota composition and its activity and also have direct effects the immune response. The newest member of the biotics family, postbiotics, refers to bioactive compounds produced by food-grade microorganisms during a fermentation process. Postbiotics include microbial cells, cell constituents and metabolites.

**Figure 1:**
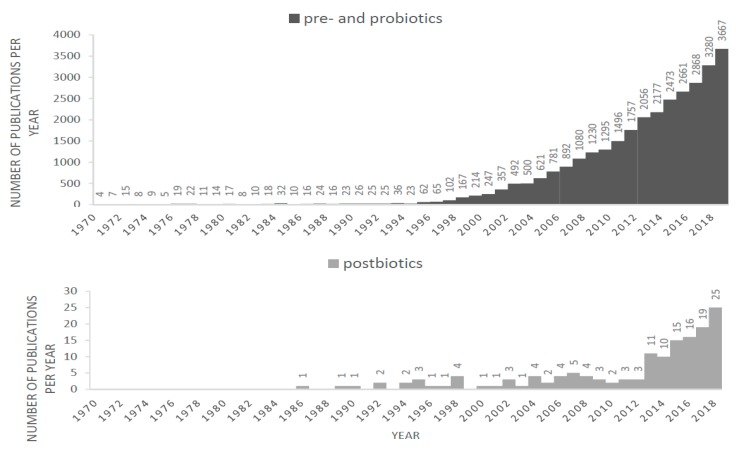
Comparison between the number of publications; on top the search of prebiotics and probiotics and below the search of postbiotics, paraprobiotics and fermented infant formulas (FIFs), and their synonyms in PubMed January 2019.

As an indication of the growing interest in nutritional strategies to modulate the gut microbiota, the number of papers reporting research in probiotics and prebiotics shows a steep increase over the past 40 years. Since 5 years reports on postbiotic products are emerging. In many of these studies the term ‘postbiotics’ is used, some studies describe applications termed ‘paraprobiotics’, ‘non-viable microbial cells’ and ‘fermented infant formulas’ (FIFs) that fit the definition of postbiotics. These terms, and their synonyms started to appear after 1986 and the use of these terms is increasing as shown in Figure 1.

### 1.1. Probiotics

Based on a 2001 Food and Agriculture Organization of the United Nations – World Health Organization (FAO-WHO) expert group consensus statement, probiotics, can be defined as ‘live microorganisms, which when administered in adequate amounts, confer a health benefit on the host’ [8,9]. The majority of the probiotic products contain a defined and limited list of microbial taxa, which mostly include lactic acid bacteria (LAB) such as; *Lactobacillus* spp. and *Bifidobacterium* spp., which have the status of being generally regarded as safe (GRAS) [10]. Notably, health effects of probiotics are very strain and disease specific, as reviewed elsewhere [11,12,13,14]. As such, evidence on each of them might be different and there is a tremendous amount of data on specific probiotic strains accompanied by a large diversity in study outcomes.

Ideally, probiotics should be physiologically and genetically characterized, and should be able to arrive in a viable state in the gut after product processing, storage conditions and gastric passage. Additionally, the health effects should be demonstrated in human studies.

It has been suggested that probiotics can influence the gut microbiota through the suppression and inhibition of pathogens as well as preventing adhesion and establishment of these pathogens in the gut [11,15]. Furthermore, probiotics may have a role in immune system development, synthesis of important nutritional elements such as vitamins, and the reinforcement of the intestinal barrier integrity through the upregulation of genes involved in tight junction signaling [12].

For the probiotic members of the genera, *Lactobacillus*, *Bifidobacterium* and *Streptococcus*, immunomodulatory properties have been shown, with beneficial effects on cell-mediated immunity and inflammation [16]. Probiotic modulation of immune development represents a promising application. Particularly in young infants where the most pronounced immune-modulating effects have been documented [17]. As such, probiotics in infant and young child nutrition have shown promising results for the management and treatment of allergies, gut and respiratory infections, irritable bowel syndrome (IBS), ulcerative colitis (UC) and infant colic. However, probiotics require further confirmation and should be used with caution in immunocompromised or severely ill children [18].

Clinical efficacy and prophylactic effects of probiotics has also been suggested in adults for several conditions, including antibiotic-associated diarrhea, acute gastroenteritis, IBS, UC and acute respiratory infections [19]. Moreover, probiotic use has been associated with a variety of immunomodulatory effects [16,20].

Changes in composition and functioning of the gut microbiota as a result of probiotic use is less clear, although some studies report that probiotics induce alterations in gut microbiota composition that co-occur with health-promoting effects [21]. However, due to a lack of experimentally demonstrated causal relationships, it is difficult to claim that such microbiome alterations are indeed beneficial [19].

### 1.2. Prebiotics

The International Scientific Association of Probiotics and Prebiotics recently reviewed the definition and scope of prebiotics, and produced a consensus statement on the definition of prebiotics: ‘a substrate that is selectively utilized by host microorganisms conferring a health benefit’ [22]. Prebiotics can change the microbiota composition by stimulating the growth of certain species, thereby promoting health benefits in the host [13].

Numerous fermentable carbohydrates have been reported to convey such prebiotic effects, including human milk oligosaccharides (HMOs), several dietary fiber types, phenolics and phytochemicals, conjugated linoleic acid and polyunsaturated fatty acids and a wide range of oligosaccharides, with wide ranges of health effects as elegantly described elsewhere [22]. The well-studied and most frequently used prebiotic oligosaccharides include short-chain galactooligosaccharides (scGOS) and long-chain fructooligosaccharides (lcFOS) [23,24,25,26,27]. The main effects of many of these prebiotics are based on the enhancement of the growth and activity of specific *Bifidobacterium* spp., which relative abundances are characteristic for breastfed infants and young children [28]. Via this microbiota modulating effect, prebiotic oligosaccharides in infant formula are known to induce changes in gut metabolic activity and bring stool consistency and frequency closer to that of breast-fed infants [27]. Besides effects in babies, prebiotics are associated with physiological and pathophysiological properties throughout life, including toddlers, adolescents, adults and elderly. These effects include, amongst others, improvement of gastrointestinal functioning and barrier function, increase in mineral absorption, modulation of energy metabolism and satiety and reducing the risk of intestinal infections [16,29].

### 1.3. Synbiotics

Synbiotics are often defined as ‘synergistic mixtures of probiotics and prebiotics that beneficially affect the host by improving the survival and colonization of live beneficial microorganism in the gastrointestinal tract of the host’ [8,9]. Synbiotics can modulate the gut microbiota composition and modulate microbial metabolite production [30]. Infant formula with added synbiotics have shown to support normal growth in infants with cow’s milk allergy, modulate the intestinal microbiota and prevent asthma-like symptoms in infants with atopic dermatitis [31,32,33]. On top of this, it was shown that infant formula supplemented with synbiotics containing scGOS/lcFOS and *Bifidobacterium breve* M-16V compensates the delayed *Bifidobacterium* colonization documented for C-section born infants. In C-section born infants these synbiotics modulate the production of acetate and the acidification of the gut. These observed physiological conditions, described as indicators of gut health, emulate the ones observed in vaginally born infants [34]. With regards to adults, several meta-analyses suggest positive effects of synbiotics in constipation, on lowering of high fasting blood glucose levels and on the risk of developing postoperative sepsis after gastro-intestinal surgery [35,36,37].

### 1.4. Postbiotics

It is important to note that the gut microbiota composition varies between populations and even individuals. The composition of the gut microbiota is linked to its metabolic and functional phenotype. Therefore, the extent to which components are microbially metabolized may differ between individuals [38]. This can lead to differences in health effects of these ‘-biotics’ between individuals [11]. Not only differences in effects can be found between specific ‘-biotics’, or between individuals, but also temporal changes in gut microbiota composition could influence the response to interventions. Moreover, many of the proposed health effects, of the addition of probiotics, prebiotics or synbiotics rely on the eventual production of short-chain fatty acids (SCFAs) and components like microbial fractions, functional proteins, secreted polysaccharides, extracellular polysaccharides (EPS), cell lysates, teichoic acid, peptidoglycan-derived muropeptides and pili-type structures [14,26,39,40,41,42].

These insights contributed to a reappreciation of food fermentation and gave rise to the postbiotics concept. Postbiotics are functional fermentation compounds, like the ones mentioned above, that can be used in combination with nutritional components to promote health. Two commonly mentioned types of postbiotics are paraprobiotics and FIFs. Paraprobiotics, or ghost probiotics, non-viable probiotics or inactivated probiotics, are now often defined as ‘non-viable or inactivated microbial cells, which, when administered in sufficient amounts confer benefits to the host’ [43,44]. Where FIFs are infant or follow-on formula that have been fermented with lactic acid-producing or other bacteria and in most cases do not contain viable bacteria [45,46].

Postbiotics may pave the way toward increasing the potency of active microorganisms or turn them into functional ingredients. Besides that, postbiotics circumvent the technical challenge of colonization efficiency and keeping the microorganisms viable and stable in the product at a high dose. This facilitates delivering the active ingredients at the desired location in the intestine, improves shelf-life, and may simplify packaging and transport [47]. Postbiotics can also be used in situations in which it is harder to control and maintain production and storage conditions such as in developing countries. Moreover, it has been suggested that using postbiotics could be an attractive alternative for other ‘-biotics’ in critically ill patients, young children and premature neonates [48,49]. The postbiotics concept may bring food, microbiology and personalized treatment even closer together [50]. Here we review the currently known postbiotic compounds, their proposed mechanisms, effects on microbial community interactions, application and clinical evidence and elaborate on future perspectives.

## 2. The Impact of Postbiotics on Host-Microbiota Interactions

Probiotics are viable by definition and their stability and viability were considered to be an essential prerequisite for their health benefits [51]. For postbiotics, however, viability is no longer the most important criterion. Postbiotic efficacy is based on the microbial metabolites, proteins, lipids, carbohydrates, vitamins, organic acids, cell wall components or other complex molecules that are generated in the matrix that is fermented [42,44]. In some cases the postbiotic composition can be influenced by food processing methods such as heat, sonication, irradiation and high pressure [52]. The microorganisms involved in the fermentation process might respond differently to these methods. For example, some proteins originating from bacteria that are inactivated by heat might denature, while irradiation might cause mutations in nucleic acids [52]. Therefore, the composition of the postbiotic product and thus the host response to the postbiotic products depends on the complete food production process [43].

The molecular mechanisms underlying the effects of postbiotics seem to be mediated through an interaction between the host and microbial products. This in turn can trigger the host immune system, and thereby trigger e.g., anti-inflammatory responses [53]. Studies describing these molecular mechanisms are often performed in vitro, mechanisms of action leading to these benefits in humans have not been fully elucidated [52]. An example of a possible mechanism immunomodulation by postbiotics in humans could be derived from an in vitro experiment showing the innate response of macrophages to non-viable *Lactobacillus casei* cells. A suspension with heat-killed bacterial cells resulted in an increase in the expression of pro-inflammatory cytokines and enhanced the transcription of Toll-like receptors (TLR-2, TLR-3, TLR-4 and TLR-9) [54]. Moreover, several in vitro studies have shown that heat-treated *Bifidobacterium* cells induce cellular immune and anti-inflammatory responses by inhibiting IL-8 secretion in intestinal epithelial cells obtained from patients with UC [55,56]. It was suggested that these effects in UC patient derived cells are induced by released microbial soluble anti-inflammatory factors that inhibit IL-8 secretion in intestinal epithelial cells. This was not caused by one single factor [56]. Furthermore, it is hypothesized that postbiotic compounds from *Lactobacilli* spp. can exert immunomodulation activity by increasing levels of Th1-associated cytokines and reducing Th2-associated cytokines [52].

Mouse experiments with fermented infant formula containing postbiotics derived from *Bifidobacterium breve* C50 and *Streptococcus thermophilus* 065 have demonstrated prolonged dendritic cell survival and maturation, and induced high IL-10 production through TLR-2, suggesting immune regulatory functions. Moreover, postbiotics from these strains have been shown to improve the epithelial barrier function and stimulate Th1 response in mouse models highlighting the involvement of postbiotic components in host immune function [57,58]. Another study using mouse models showed that metabolic products of fermented infant formula by *Lactobacillus paracasei* CBA L74 could act via the inhibition of immune cell inflammation and protect the host from pathobionts and enteric pathogens and have protective effects against colitis [59].

Two other, well described, fermentation products associated with health benefits are exopolysaccharides (EPS) and extracellular vesicles (EVs) [60,61]. A broad range of bacterial taxa has the capability to synthesize these EPS polymers, including *Bifidobacterium* species [62,63]. Two types of EPS can be distinguished, homopolysaccharides (HoPS) and heteropolysaccharides (HePS). This classification depends on the composition of the repeating units, where HoPS consist of one type monosaccharide and HePS consist of two or more types of sugars [64]. Several health benefits of EPS have been described, such as cardioprotective, antiulcer, antioxidant and cholesterol lowering effects [65,66]. Furthermore, EPS from *Lactobacillus plantarum* 70810 was found to function as antitumor agents in vitro by inhibiting the proliferation of HepG-2, BGC-823 and HT-29 tumor cells [67]. However, human clinical trials are needed to assess the safety and health promoting efficacy of different forms of microbial EPS [60].

The EVs, as mentioned earlier, are spherical lipid bilayer structures that can be secreted by both Gram-negative and Gram-positive bacteria [68]. Moreover, EVs carry a large diversity of compounds such as proteins, nucleic acids, phospholipids, glycolipids and polysaccharides [61]. Two types of EVs can be distinguished; outer membrane vesicles (OMVs) for Gram-negative bacteria and membrane vesicles (MVs) for Gram-positive bacteria [61]. EVs have multiple potential biological functions, ranging from roles in the microbial community interactions, e.g., transferring genetic material and proteins, to host-microbe signaling [69]. Several studies can be found that show a positive effect of EVs on host cells. For example, *Akkermansia muciniphila* and commensal *Escherichia coli* derived EVs have shown respectively to decrease gut permeability and activate signaling through the intestinal epithelial barrier in vitro [69,70]. Some studies investigated the effect of EVs in vivo, indicating possible effects of EVs in the protection against UC in mice [71]. However, similar to the postulated effects of EPS, human clinical trials are needed to establish safety and potential for the use of EVs as therapeutic agents in humans.

Despite the limited amount of studies on specific microbial compounds in postbiotics such as organic acids, EPS and EVs, several human intervention studies have been conducted investigating the beneficial effect of postbiotics. Available literature on clinical studies reporting application of fermented infant formulas containing postbiotics, are reviewed in more detail below.

### 2.1. Human Intervention Studies with Postbiotics in Early Life, Including New-Borns, Infants and Toddlers, until Adulthood (<18 Years)

An overview of studies in which the effects of postbiotics, mostly FIFs, were investigated can be found in Table 1 [46,72].

#### 2.1.1. Studies Using *B. breve* C50 and *S. thermophilus* 065 Combined with Prebiotics (scGOS/lcFOS)

Clinical evidence for the use of a FIF with different amounts of fermentation product derived from *B. breve* C50 and *S. thermophilus* 065 combined with prebiotics (scGOS/lcFOS), suggests that this product is safe to use and well tolerated in healthy term newborns. No adverse events (AEs) were found [73,74,75]. For example, the potential effect of FIF containing *B. breve* C50 and *S. thermophilus* 065 (scGOS/lcFOS on newborns aged 0–28 days with colic was investigated [73]. It was demonstrated that the product had effects against infantile colic, by reducing overall crying time and stool softening [73]. This was not associated with differences between daily weight gain for all groups, confirming that a FIF with scGOS/lcFOS is safe to use and results in normal growth in healthy newborns [74]. Moreover, this study underlines the beneficial effects of prebiotic scGOS/lcFOS on gut microbiota composition and stool characteristics, irrespective of the presence or dose of the fermented formula (none, 15% and 50%) [74]. Another study compared newborns aged 0–28 days consuming FIF for the effects on stool consistency. It was reported that in case of the FIF consumption, 30% fermented product with scGOS/lcFOS, stool consistency was significantly softer than standard formula and closer to that of breast-fed infants and [75]. These results are promising, but also show that studies taking dose-response into account are key, as different concentrations of the fermented formula (0%, 15%, 30% and 50%) can make a difference. Interestingly, beneficial effects on stool consistency was also observed in the scGOS/lcFOS only group [74]. Thus, scGOS/lcFOS was attributing to the result while the effect of the combination with fermented formula was not clear.

#### 2.1.2. Studies Using *B. breve* C50 and *S. thermophilus*

Studies investigating the clinical effects of formula containing postbiotics from *B. breve* C50 and *S. thermophilus* suggests that this product is safe to use, results in normal growth and were well tolerated in newborns, term and preterm and infants [76,77,78,79,80,81,82]. The results of these studies ranged from differences in microbiota composition, gastrointestinal effects such as the incidence of diarrhea or lower concentrations of calprotectin but also systemic effects like antipoliovirus IgA response [79,80,81]. One study investigated the effect of such product on preterm infants aged 2–5 weeks in terms of clinical tolerance and its effect on the gut microbiota, namely Tumor Necrosis Factor α (TNF-α), calprotectin and secretory IgA (SIgA). It was reported that there was clinical tolerance and the consumption resulted in lower abdominal distention. Moreover, benefits on inflammatory and immune markers were shown, but did not show significant changes in bacterial colonization. Moreover, lower fecal calprotectin levels were found in the FIF group [76]. In contrast a study on healthy term infants of 3–7 days old reported no statistically significant differences in fecal calprotectin concentrations between the subjects receiving FIF, standard formula or breast feeding [81]. Another study investigated the effect of such product on newborns, included at birth, who were at high risk of allergy. Here it was observed that this led to a reduction of digestive and respiratory infections compared to standard infant formula. The proportion of children with cow’s milk allergy did not change but positive skin-prick test responses to cow’s milk proteins were lower in the FIF group [77]. A comparison of thymus indices, in children aged 3 days, showed no significant differences between subjects receiving FIF and breast-fed infants. However, this study found lower fecal pH in breast fed and FIF consuming infants compared to the control formula from the third postnatal day, persisting during the 4 months of the study [78]. The possible effect of the postbiotic compounds was also tested for poliovirus-specific intestinal antibody response, as the researchers hypothesized that postbiotic compounds might enhance colonization of bifidobacteria, which thereby possibly trigger an intestinal immune response. In this case positive results were found for poliovirus-specific intestinal antibody response. The rise in antipoliovirus IgA increased significantly after a Pentacoq^®^ challenge compared to the control formula. On top of this, fecal bifidobacterial levels were significantly higher in the FIF group [79].

Acute diarrhea is a prominent problem as it is one of the most common causes of death in infants and children in developing countries [83]. However, also in western countries, although most cases take a mild to moderate severity, acute diarrhea is responsible for significant morbidity with major impact on health care costs [84]. It is hypothesized that FIFs could promote bifidogenic effects as well as show immunomodulatory effects and potentially help in the treatment of acute diarrhea. In relation to the two other studies mentioned above, that showed positive effects of this FIF to SIgA and antipoliovirus IgA, this FIF might also enhance e.g., antirotavirus IgA, a common cause of acute diarrhea in children [76,79,80]. As such, two studies investigated the effect of this FIF on incidence of acute diarrhea and duration. Results of both studies were contradictive as no effects on incidence or duration of acute diarrhea or hospital admissions were found in children aged 4 to 6 months. However, diarrhea episodes were less severe [80]. Another study in children aged less than 5 months with diarrhea reported significantly less children with diarrhea in the FIF group compared to control infant formula [82]. These differences in outcome might be explained by the fact that the first study investigated acute diarrhea, while the later investigated the incidence of diarrhea in children at early weaning.

#### 2.1.3. Studies Using Other Postbiotic Products

Several other studies can be found that investigated the use of different types of postbiotic products.

Some of these studies investigated the effect of postbiotic products on diarrhea in children below the age of 4 years, showing mixed findings indicating that the use of postbiotics in children with diarrhea should be done with great care. One study in children aged below 24 months with non-rotavirus diarrhea investigated the effect of heat-killed *L. acidophilus* LB plus its culture medium added to oral rehydration solution (ORS) compared to ORS only. In this study, recovery time was shortened by 1 day in the intervention group compared to ORS only. Beside clinical evidence, this study also investigated the effect of the postbiotic product on fluid-formed domes, a measure for anti-secretory activity, in cultured human intestinal Caco2/TC7 cell monolayers, infected with *E. coli* C1845 bacteria and found that the postbiotic products antagonizes the C1845-induced increase in paracellular permeability [85]. Another study, investigating the effect of heat inactivated *L. casei* strain GG on rotavirus diarrhea in in children below the age of 4 years, did not show such clear positive effects of the postbiotic compared to the viable *L. casei* strain. With regards to the duration of diarrhea, no significant differences were found between groups. However, significant differences at convalescence were found with rotavirus specific IgA secreting cells in the viable *L. casei* group, compared to the postbiotic group [86]. Another study investigated the effect of micronutrients (including zinc) with or without heat inactivated *L. acidophilus* compared to a placebo in infants aged 6–12 months at high risk for diarrhea related mortality (defined as at least one episode of diarrhea in the preceding two weeks). This study focused on the longitudinal prevalence of diarrhea, which was lowest in the intervention group without heat inactivated *L. acidophilus* (micronutrients only), while the intervention group with heat inactivated *L. acidophilus* showed more days of diarrhea and fever, although not significantly different from placebo. The authors therefore conclude that the intervention with heat inactivated *L. acidophilus* has a negative effect in these children [87]. In relation to this, a study assessing the efficacy of viable and heat-inactivated *L. rhamnosus* strain GG in infants, mean age 5.5 months, in the management of atopic eczema and allergy to cow’s milk. Although the authors did not report significant differences between groups in symptom scores or differences in bacterial numbers within the genera enumerated, the treatment arm with heat-inactivated *L. rhamnosus* GG was associated with adverse gastrointestinal symptoms and diarrhea. Therefore, the study for this arm was terminated after the pilot due to these adverse events [88]. Therefore, due to the mixed findings and even contradictory outcomes, the use of postbiotics in children with diarrhea should be done with great care.

Two studies showed an effect of a postbiotic cow’s skim milk fermented with *L. paracasei* CBA L74 on common infectious disease (CID) in toddlers aged 12–48 months compared to a placebo. This study indicates that this product can prevent common infectious diseases in toddlers in terms of the proportion of children presenting with CID, acute gastroenteritis and upper respiratory tract infections [89]. Moreover, via random selection of a subset of the above mentioned study, the effect of cow’s skim milk fermented with *L. paracasei* CBA L74 in toddlers aged 12–48 months on the overall gut microbiota was investigated. It was found that the relative abundance of *Lactobacillus* and *Ruminococcaceae* was increased in the intervention group with specific significant increases in *Oscillospira* and *Faecalibacterium*. At the subgenus level, *Roseburia* increased after intervention and showed positive correlative associations with SIgA and β-defensin. *Blautia* and *Bacteroides* also increased, and showed positive correlative associations with α-defensin. Furthermore, the intervention showed an increase in the relative abundance of predicted genes involved in butyrate synthesis, especially genes encoding butyryl coenzyme A transferase and butyrate kinase. Moreover, increased fecal butyrate levels in the intervention group [90].

Two other studies showed that a postbiotic product has similar effects as a product containing live bacteria. One study on live and heat-killed *L. paracasei* 33 in children aged below 18 years investigated the effects on allergic rhinitis induced by house-dust mite. It was reported that the product could effectively improve the overall quality of life as measured by a modified pediatric rhino conjunctivitis quality of life questionnaire (PRQLQ) containing five domains of nose, eye, other symptoms, practical problems and activity limitations, compared to a placebo product. For the heat-killed *L. paracasei* 33 group, significant improvement was found in PRQLQ for nose, other symptoms, total score for overall quality of life, level of bother due to nose symptoms and practical problems (all *p* < 0.05). Moreover, it was found that heat-killed *L. paracasei* 33 was not inferior to a product containing live *L. paracasei* 33 cells [91]. In line with this, another study found that a postbiotic product was not inferior to the live probiotic in a study in children aged 10–12 years with lactose malabsorption. This study investigated the effect between live (Lacidofil) and heat-killed (Dialac) probiotics on lactose malabsorption by a breath hydrogen test (BHT) and indicated no differences between both groups. However both groups showed a significant decrease of BHT results after 120 min after giving the investigated products, indicating less lactose malabsorption. Thus, indicating that both live and heat-killed probiotics can decrease BHT in children with lactose malabsorption [92].

### 2.2. Human Intervention Studies with Postbiotics in Later Life, Including Adults and Elderly (>18 years).

An overview of studies that investigated the effect of postbiotics later in life, including adults and elderly (>18 years) can be found in Table 2.

All studies using postbiotics in adults are products derived from non-viable lactobacilli species. These studies suggest that inactivated lactobacilli species are safe to use. Moreover, most studies found positive effects for the use of postbiotics on diverse primary outcomes, especially for gastrointestinal functioning and in the treatment of chronic diarrhea or diarrhea predominant IBS [93,94,95].

One study investigated the effect of postbiotic *L. paracasei* K71 versus a placebo in adults, aged 20–65, on atopic dermatitis. Skin severity scores were significantly improved after 8 and 12 weeks in the intervention group, but not in the placebo group. Consumption of topical therapeutics, though not significant, was 1.9 times more in the placebo group compared to the intervention group. However, differences between groups on itch scores and quality of life did not show such clear effects [96].

Several studies focused on potential protective effects of postbiotics on immune responses. Overall, despite the fact that these products seem safe to use in adults, these studies do now show very clear evidence to use postbiotic products in the protection against human rhinovirus (HRV) or for influenza vaccinations. However, in elderly over the age of 65 postbiotic *L. pentosus* b240 can reduce, dose-dependently, the rate of common cold, as shown by a study investigating the effect of postbiotic *Lactobacillus pentosus* b240 in a low or a high dose in elderly over the age of 65 compared to a placebo on the incidence of common cold. The high dose (2 × 10^10^ heat-killed cells) showed the lowest accumulated incidence rate, as well as the highest quality of life, followed by the low dose (2 × 10^9^ heat-killed cells) and last the placebo. This indicates not only that this postbiotic might be effective in the prevention of common cold infections, but that there is a dose-response effect as well [97]. Less clear indications for the positive effects of postbiotics were found in a study in women (average age 45.4 ± 8.1 years) investigating the effects of *L. plantarum* L-137 versus placebo on interferon (IFN) response after trivalent inactivated influenza vaccine. No differences were found in seroresponse rate, seroprotection rate and geometric mean Ab titers after the first or second dose of vaccine. However, IFN-β levels were significantly higher compared to the placebo group [98]. Likewise, no clear positive effects of a postbiotic were found in the most recent study, that investigated the effect of juice enriched with postbiotic *L. rhamnosus* GG in adults aged 18–65 years, compared to juice enriched with live *L. rhamnosus* GG or a placebo after a tissue culture infectious dose of HRV. This study showed no significant differences in viral loads, but reported a tendency towards the lowest HRV load in the postbiotic group [99].

With regards to the effects of postbiotics on different causes of diarrhea and gastrointestinal functioning, the use of postbiotics appears to be safe and studies with postbiotics show positive effects of the postbiotic product, in contrast to the studies mentioned above in children. One study investigated the effect of heat-killed *Lactobacillus acidophilus* LB in adults, 16 years and over, compared to living *L. acidophilus* LB on chronic diarrhea. From the second week onwards, stool frequency was significantly lower in the postbiotic group, as well as improvement in clinical symptoms. This indicates that the postbiotic product was more effective than the living *L. acidophilus* LB in the treatment of chronic diarrhea [95]. In line with this, another study investigated the same product of heat-killed *L. acidophilus* LB in adults (mean age 53.4 ± 17.3 years) with diarrhea predominant IBS. This study did not have any control group, but only compared the baseline data to the data after 1 month of consuming the postbiotic, where pain scores, bloating and quality of life improved after treatment [94]. This indicates that postbiotics, especially *L. acidophilus* LB might be a promising intervention in the treatment of diarrhea, however more randomized controlled trials are needed to confirm this effect in adults. Related to this, another study investigated the effect of *Lactobacillus gasseri* CP2305 versus a placebo on gastrointestinal function in healthy adults, of 20–70 years of age, with a tendency towards constipation or with frequent bowel movements. Subjective and objective Bristol stool scale scores, output and color tone were improved in the postbiotic group, especially in the subgroup of people with a tendency towards constipation. The postbiotic had a beneficial effect on the regulation of intestinal function. Moreover, this study also analyzed fecal samples and showed that SCFAs (i.e., propionic acid, butyric acid and valeric acid) and *Clostridium* cluster IV were significantly increased in the postbiotic group, but not in the placebo group [93]. Even though the mechanisms underlying the improved regulation of intestinal function remain unclear, this study indicates that non-viable probiotic bacteria and their fermentation products, the postbiotics, can have clear clinical effects as well as induce changes in SCFAs and gut microbiota composition. Possible mechanisms underlying these effects and the role of postbiotics on microbial community interactions will be discussed below.

## 3. Effects of Postbiotics on Microbial Community Interactions

Postbiotics can have direct and indirect effects on the composition and functioning of the human gut microbiota. Fermentation products, such as organic acids, could inhibit the growth and activity of potential pathogens but could also be utilized by specific microbial taxa in the gut that in their turn can synthesize SCFAs [90]. Below direct and indirect effects of several separate postbiotic compounds will be discussed.

As mentioned earlier in this review, major end products of gut microbiota activity are SCFAs. These can be present in postbiotic products, leading to direct effects of these SCFAs or to microbial cross-feeding. With regards to direct effects, the primary produced SCFAs are acetate, propionate and butyrate [100]. The primary SCFAs are known to stimulate colonic sodium and fluid absorption and were also found to exert proliferative effects on colonocytes. The most abundant SCFA detectable in human peripheral circulation is acetate, as propionate is metabolized by the liver being a major substrate for gluconeogenesis, and butyrate is absorbed and used as the primary source of energy by colonocytes [101,102]. For this reason, of these produced SCFAs, butyrate has been investigated most extensively. Moreover, butyrate is associated with multiple health benefits e.g., butyrate was found to enhance the intestinal barrier function and mucosal immunity as reviewed elaborately elsewhere [103,104,105]. In addition, butyrate and to a lesser extent propionate are known to act as histone deacetylase (HDAC) inhibitors. Histone acetylation is used to increase accessibility of the transcriptional machinery to promote gene transcription; acetyl groups are removed by these HDACs. By doing so, they exert anti-inflammatory and immune effects through suppression of lamina propria macrophages and cause differentiation of dendritic cells from bone marrow stem cells [101,106,107,108]. SCFAs can also modulate cellular activity extracellularly through SCFA-specific G-protein coupled receptors (GPRs) present on e.g., gut epithelial cells, among others [109]. In terms of health and disease, SCFAs have also been linked to for example anti-tumor effects, anti-inflammatory effects on the colonic epithelium, protection from development of immune disorders, control of obesity, control of glucose homeostasis, appetite regulation and cardiovascular effects as extensively reviewed elsewhere [109,110].

With regards to cross-feeding on SCFAs, the pathways for biosynthesis of SCFAs from the fermentation of indigestible fibers support a bacterial cross-feeding complex that involves several SCFA synthesis pathways to produce acetate, propionate and butyrate [39,101]. These interactions can only take place due to the enzymatic repertoire of specific members of the gut microbiota. Acetate is produced both via the fructose-6-phosphate phosphoketolase (F6PK) pathway, commonly referred to as the bifid shunt, and the Wood-Ljungdahl pathway from pyruvate via acetyl-CoA by e.g., *Blautia hydrogenotrophica*, *Clostridium* and *Streptococcus* spp. [111]. Propionate can be produced via three main pathways e.g., *Bacteroides* spp. and *Roseburia inulinivorans* generate propionate via the acrylate pathway via pyruvate after which lactate is reduced to propionate. *Bacteroides fragilis* uses the ‘succinate pathway’ by which phosphoenolpyruvate (PEP) or pyruvate is used to produce succinate and later propionate. Last, members of Lachnospiraceae, including *R. inulinivorans* and *Blautia* species can generate propionate and propanol from the deoxy-sugars rhamnose and fucose via the propanediol pathway via propionyl-CoA [111,112]. Butyrate is synthesized from two molecules; acetyl-CoA, which is converted into butyryl-COA via β-hydroxybutyryl-CoA, and crotonyl-CoA [101]. Characteristic examples of butyrate producing members of the gut microbiota are *Faecalibacterium prausnitzii*, *Eubacterium rectale*, *Roseburia intestinalis* and *Anaerostipes* spp. [113].

Next to cross-feeding on SCFAs, several studies have also focused on cross-feeding on micronutrients, such as B-group vitamins, part of which essential in all microbes and the mammalian host [114,115]. These precursors of indispensable metabolic cofactors can be produced by some gut bacteria, but many other gut bacteria as well as the mammalian host do not have the capability to produce B-group vitamins [114]. Recently, a study using humanized gnotobiotic mice and in vitro anaerobic fecal culture showed that B-vitamin exchange and sharing may have a strong contribution to stability of gut microbial communities [115].

For another postbiotic compound, EPS, not only direct effects for the host can be observed. Postbiotic EPS-like compounds may also play a role in the modulation of the gut microbiota composition and activity. Some EPS polymers can be used as fermentable substrates by commensal gut bacteria because these carbohydrate polymers are composed of one HePS, two or more HoPS types of sugars. Thereby promoting the production of metabolites benefitting the host [60,116]. An example of cross-feeding on EPS has been described for some *Bifidobacterium* strains. EPS produced by *Bifidobacterium* strains can act as fermentable substrates in vivo and thereby lead to shifts in metabolite production profiles and interactions between gut bacteria [117]. Another study showed that EPS produced by a marine LAB named *Weissella cibaria* has a strong bifidogenic effect in vitro [66]. Similarly, EPS produced by *L. plantarum* has been shown to promote the growth of beneficial species like *B. longum* and *L. acidophilus* [65]. However, it is important to note that no human intervention studies have been performed to confirm these effects on the microbial community interactions described in in vitro studies.

Lastly, postbiotic compounds might play a role in the inhibition of pathogens. The postbiotic components responsible for pathogen inhibition are likely to be bacteriocins or organic acids [118]. Bacteriocins are ribosomally synthesized antimicrobial peptides, exhibiting bacteriostatic or bactericidal properties [118,119]. For example, probiotic products from six different *L. plantarum* strains were found to inhibit both gram-positive and gram-negative pathogens [118].

Even though adult human intervention studies reporting changes in microbiota in using postbiotics are scarce, one study showed significant increase in propionic acid, butyrate and valeric acid and a significant increase in *Clostridium* cluster IV [93]. Moreover, a few studies report on the influence of fermented formula on the infant gut microbiota [74,76,78]. In these studies it was noted that the fecal pH decreased upon supplementation of fermented formula [74,78]. In addition, high relative amounts of acetate were measured [74]. Even though these studies did not focus on the gut microbiota composition directly, these decreases in pH could indicate an increase in SCFA production and modulation of the gut microbiota composition towards SCFA production, which may be beneficial to the host.

Altogether, specific postbiotics may have a role in the modulation of the gut microbiota and thereby benefitting host health, by favoring the growth of beneficial microbial species, as well as inhibiting the growth and activity of potential pathogens.

## 4. Discussion and Future Perspectives

Studies in the past sometimes used heat-inactivated probiotics as a control for the effect of probiotics. However, this review emphasizes that non-viable microbial cells or any postbiotic should not be considered a control, as they can have direct immunomodulatory and clinically relevant effects. As shown in this review, several studies showed clinically relevant or immunomodulatory effects of postbiotics.

This review describes examples of postbiotics including FIFs and inactivated cells that can modify biological responses. We therefore think that postbiotics fit well among the other ‘-biotics’ family members. The biological responses of postbiotics have been observed in cell cultures, animal models and are confirmed by the human trials discussed in this review [52]. However, in order for postbiotics to be widely accepted as functional foods with substantiated health-benefits an expert consensus on the exact definition of postbiotics is needed, as was formulated by e.g., ISAPP, WHO or FAO for prebiotics and probiotics [8,9,22]. This is of high importance not only for clarity within the field of microbiology or nutrition, but also from a regulatory perspective. Even more so, because 1) postbiotics can be made with a wide range of probiotic species, 2) postbiotics can be obtained via a wide range of inactivation methods, 3) postbiotics may differ in delivery method and shelf life and 4) appropriate methodologies should be used to evaluate biological and clinically relevant effects [52]. Only with a clear definition of postbiotics, health benefits can be linked to (subcategories of) postbiotics. We proposed the following working definition for postbiotics: Postbiotics are bioactive compounds produced by food-grade micro-organisms during a fermentation process. Postbiotics include microbial cells, cell constituents and metabolites.

Evidence can be found for the use of postbiotics in healthy children for a range of diseases or to improve general health, however especially in children with diarrhea this should be done with care due to the inconsistent observations in studies investigating different causes of diarrhea [80,82,85,86,87,88]. These findings are in contrast to the findings for diarrhea in adults, as in these studies the postbiotics seems to be favorable in the treatment of diarrhea [94,95]. Moreover, results of some studies need to be evaluated with caution, as some products used contained or may contain prebiotics.

Another issue we want to underscore is that most studies described in this review did not take microbiota composition into account. Insight into the impact on host-microbe interactions is vital to understand the potential benefits and risks of postbiotics. Therefore, not only gut microbiota composition or measurements of e.g., SCFAs are important parameters for future (clinical) studies, but also more holistic approaches including metatranscriptomics, metaproteomics and metabolomics will contribute to a better understanding of these complex interactions between postbiotics, host biology and the gut microbiota and will give a better insight in the mechanisms underlying health effects of postbiotics.

In conclusion, postbiotics may contribute to the improvement of host health, even though the exact mechanisms have not been fully elucidated. In addition to mechanism of action focused preclinical and in vitro studies, well-designed randomized placebo-controlled intervention studies are needed to demonstrate health effects of postbiotics. Moreover, due to advances in measuring the composition and function of the gut microbiome we enter a new era of ‘-biotic’ research. This contributed already and will continue to contribute to extend the range of compounds with potential health benefits that can be applied in specialized nutrition. Ideally, advances in gut microbiota research will contribute to specifically design individual recommendations in terms of personalized nutrition or interventions to improve health. Postbiotics can be an elegant and safe method to improve health as postbiotics have less challenges compared to viable probiotics in terms of storage and shelf-life. Moreover, as shown in this review, several studies show comparable results for the viable probiotic and the postbiotic product and might be a safer alternative to probiotics in immunocompromised or severely ill children [18,91,92]. Furthermore, postbiotics and bioactive compounds may be an effective way to increase the potency of probiotics to turn them into functional ingredients or therapeutic agents [53].

## Figures and Tables

**Table 1:** Studies reporting interventions with postbiotics in early life, including newborns, infants and toddlers, until adulthood (<18 years).

Study	Type of study	Intervention	Comparison	*N* (age at start)	Population	Duration	Outcome
Berni Canani et al. 2017 [90]	Double-blind, randomized, controlled trial	Cow’s milk powder Fermented with *L. paracasei* CBA L74	Cow’s milk powder with maltodextrin	20 (12–48 months)	Healthy, term children	3 months	Based on 16S rRNA gene amplicon sequencing of fecal samples, the relative abundance of *Lactobacillus* was increased and individual *Blautia*, *Roseburia* and *Faecalibacterium* oligotypes were associated with the intervention but not the placebo, showing correlative associations with immune biomarkers. The intervention, not placebo, showed an increase in the relative abundance of predicted genes involved in butyrate synthesis by PICRUSt-predicted metagenomes.
Corsello et al. 2017 [89]	Double-blind, randomized, controlled trial	Cow’s Milk Fermented with *L. paracasei* CBA L74	Cow’s milk with maltodextrin	146 (12–48 months)	Healthy, term children	3 months	Children presenting common infectious diseases were significantly lower in the intervention group compared to the placebo group in both intention to treat (60% vs. 83%, absolute risk difference of 23%, *p* < 0.01) and per protocol (18% vs. 40%, absolute risk difference of 22%, *p* < 0.01). Moreover, significant changes in innate and acquired immune biomarkers were only observed in the intervention group.
Vandenplas et al. 2017 [73]	Double-blind, randomized, controlled trial	Lactofidus 50%FERM, scGOS/lcFOS+ 15%FERM or scGOS/lcFOS+ 50%FERM	scGOS/lcFOS	432 (0–28 days)	Healthy, term infants	17 weeks	All formulas were well tolerated, infant colic was significant lower (8%) with scGOS/lcFOS+50% FERM than scGOS/lcFOS (20%, *p* = 0.034) or 50% FERM (20%, *p* = 0.036) at week 4. Daily crying duration was lower in the scGOS/lcFOS + 50%FERM and stools were softer compared to 50% FERM.
Huet et al. 2016 [74]	Double-blind, randomized, controlled trial	Lactofidus 50% FERM, scGOS/lcFOS+ 15% FERM or scGOS/lcFOS+ 50% FERM	scGOS/lcFOS	432 (0–28 days)	Healthy, term infants	17 weeks	Equivalence of weight gain (SD) per day in all groups, scGOS/lcFOS 29.7 (6.1), scGOS/lcFOS+ 15% FERM 28.5 (4.8), scGOS/lcFOS + 50% FERM 28.5 (5.0) and 50% FERM 28.7 (5.9) g/day. No differences in other growth parameters, formula intake or number/severity of AEs. All scGOS/lcFOS-containing formula; lower stool pH and *Clostridium difficile* levels and higher IgA levels.
Herrera et al. 2015 [75]	Double-blind, randomized, controlled, trial Meeting abstract	Lactofidus scGOS/lcFOS+ 30%FERM	scGOS/lcFOS and *N* = 100 breast-fed children as reference group	200 (0–28 days) 100 reference group	Healthy, term infants	17 weeks	The scGOS/lcFOS + 30% FERM was well tolerated. Stool consistency for the scGOS/lcFOS + 30% FERM group was closer to the breast-fed reference group, and a significantly softer median from 4 weeks onwards compared to the scGOS/lcFOS group (*p* ≤ 0.005). From week 9 onwards scGOS/lcFOS + 30% FERM had a significant higher median stool frequency compared to scGOS/lcFOS only (*p* ≤ 0.05).
Campeotto et al. 2011 [76]	Double-blind, randomized, controlled trial	Preterm infant formula, heat-inactivated FERM with *B. breve* C50 and *S. thermophilus* 065	Preterm infant formula	58 (0–3 days)	Pre-term infants 30-35 weeks of GA	During hospital stay; 2–5 weeks	No differences between groups in anthropometrics and digestive tolerance, except abdominal distention, which was lower in the FERM group (0 FERM vs. 8 control, *p* = 0.016). Bacterial colonization was not different between groups. Significant lower fecal calprotectin was found in the FERM group from week 3 (*p* = 0.01).
Morisset et al. 2011 [77]	Double-blind, randomized, controlled trial	Infant formula, heat-inactivated with *B. breve* C50 and *S. thermophilus*	Standard infant formula	129 (birth)	Infants with a high risk of atopy	12 months, follow-up at 24 months	The fermented formula did not alter proportion of children with cow’s milk allergy, but decreased the proportion of positive skin prick tests to cow’s milk (1.7% vs. 12.5%, *p* = 0.03), incidence of digestive AEs (39% vs. 63%, *p* = 0.01) and respiratory potentially allergic AEs at 12 months (7% vs. 21%, *p* = 0.03) and respiratory potentially allergic AEs at 24 months (13% vs. 35%, *p* = 0.01)
Rampengan et al. 2010 [92]	Pretest–posttest single blinded randomized study	Lacidofil capsules containing heat-inactivated *Lactobacillus helveticus* R-52 and *L. rhamnosus* R-11	Dialac sachets	79 (10–12 years of age)	Healthy children with lactose mal-absorption	2 weeks	Both groups showed a significant decrease before vs. after consumption of the intervention products of the BHT (from 34.51 ± 10.35 to 22.13 ± 12.41, *p* < 0.001 for live probiotics and from 36.00 ± 10.18 to 20.30 ± 8.68, *p* < 0.001 in the killed probiotics group). No differences were found between groups after intervention (*p* = 0.453).
Indrio et al. 2007 [78]	Double-blind, randomized, controlled trial	Infant formula, heat-inactivated with *B. breve* C50 and *S. thermophilus*	Standard infant formula And *N* = 30 breast-fed children as reference group	60 (3 days) 30 reference group	Healthy, term infants	4 months	Fecal pH was lower in breast-fed infants (*p* < 0.05), however it was similar to the FIF group from the third postnatal day onwards for the entire 4 months. The FIF group showed comparable thymus indices compared to breast-fed infants. Probiotic fermentation products have effects comparable to those of the bacteria composing the intestinal microbiota.
Lievin-Le Moal et al. 2007 [85]	Double-blind, randomized, controlled trial	Heat-killed *L. acidophilus* LB plus culture medium	Placebo sachets	80 (10 months)	Infants with acute diarrhea of suspected infectious origin	96 h of which 72 h with intervention products	Recovery time of infants with nonrotavirus diarrhea was shortened by 1 day when taking lyophilized, heat-killed *L. acidophilus* LB plus their culture medium (time until the first normal stool was passed compared to the placebo (39.5 ± 10.5 h vs. 63.4 ± 14.9, *p* < 0.01).
Sharieff et al. 2006 [87]	Triple-blind, randomized, controlled trial	Micronutrient sachets with heat-inactivated LAB	Micronutrient sachets or placebo sachets	75 (6–12 months)	Healthy infants with high risk for diarrhea related mortality	2 months	Prevalence of diarrhea was 26% in the micronutrient with LAB group, 15% in the micronutrient group and 26% in the placebo group; difference between the micronutrient with LAB and placebo was not significantly different.
Peng et al. 2005 [91]	Double-blind, randomized, controlled trial	Capsules with live or heat-killed *L. paracasei* 33	Placebo capsules	90 (<18 years)	Patients with perennial allergic rhinitis	30 days	QOL increased in both intervention groups, compared to the placebo in frequency (9.47 ± 2.89, 6.30 ± 2.19 vs. –3.47 ± 1.53, respectively; *p* < 0.0001) and level of bother (5.91 ± 3.21, 6.04 ± 2.44, vs. –2.80 ± 1.64, respectively; *p* = 0.004). Efficacy of heat-killed *L. paracasei* LP33 was not inferior to the live variant.
Mullie et al. 2004 [79]	Double-blind, randomized, controlled trial	Infant formula, heat-inactivated with *B. breve* C50 and *S. thermophilus*	Standard infant formula	30 (first days of life)	Healthy term infants	4 months	In the FIF group at 4 months significant higher bifidobacteria levels (*p* = 0.0498) and *Bifidobacterium longum/Bifidobacterium infantis* (*p* = 0.0399) compared to standard formula. Antipoliovirus IgA increased after Pentacoq^®^ challenge (*p* < 0.001), rise was significantly higher in the FIF group (*p* < 0.02). antibody titers correlated to bifidobacteria, especially with *B. longum/B. infantis* and *B. breve* (*p* < 0.002).
Thibault et al. 2004 [80]	Double-blind, randomized, controlled trial	Infant formula, heat-inactivated with *B. breve* C50 and *S. thermophilus*	Standard infant formula	971 (4–6 months)	Healthy term infants	5 months	Incidence and duration of diarrhea and number of hospital admissions did not differ significantly between groups. In the FIF group compared to standard formula diarrhea episodes were less severe, fewer cases of dehydration (2.5% vs. 6.1%, *p* = 0.01), fewer medical consultations (46% vs. 56.6%, *p* = 0.003), fewer ORS prescriptions (41.9% vs. 51.9%, *p* = 0.003) and fewer switchers to other formula (59.5% vs. 74.9%, *p* = 0.0001).
Campeotto et al. 2004 [81]	Prospective study	Infant formula, heat-inactivated with *B. breve* C50 and *S. thermophilus*	Standard infant formula N = 32 breast-fed	37 (3–7 days)	Healthy term infants	3 months	Fecal calprotectin concentrations did not significantly differ between groups (medians; standard formula 148 μg/g, FIF 144 μg/g and breast milk 204 μg/g), but higher (total median calprotectin 167 μg/g) than the reference value for healthy adults (50 μg/g).
Kirjavainen et al. 2003 [88]	Double-blind randomized	Infant formula containing live or heat inactivated *L. rhamnosus* GG	Hydrolyzed whey formula	35 (mean age 5.5 months)	Infants with atopic eczema and allergy to cow’s milk	Mean of 7.5 weeks	Atopic eczema and subjective symptoms decreased in all three groups and did not differ significantly between groups. No differences were found in the bacterial numbers within the genera enumerated. However, heat inactivated *L. rhamnosus* GG was associated with adverse gastrointestinal symptoms and diarrhea.
Kaila et al. 1995 [86]	Double-blind, randomized	Heat inactivated *L. casei*	Viable *L. casei* 10^10-11^ colony forming units	41 (<4 years)	Infants with acute rotavirus diarrhea	5 days	No significant differences at the acute state for specific antibody secreting cells against rotavirus between heat inactivated *L. casei* vs. viable *L. casei* (2/8 vs. 2/9, *p* = 0.66) or mean antibodies (0.1 vs. 0.04, *p* = 0.52). In contrast to the convalescent stage, in favor of the viable *L. casei* for antibody secreting cells (2/13 vs. 10/12, *p* = 0.002) and mean antibodies (22.4 vs. 50.7, *p* = 0.04). Clinical recovery from rotavirus diarrhea was equal in both groups.
Boudraa et al. 1994 [82]	Randomized study	Infant formula, heat-inactivated with *B. breve* C50 and *S. thermophilus*	Standard infant formula	84 (<5 months)	Healthy infants	Approx. 85 days	Rate of acceptance was similar in both groups. The FIF group had significantly less children with diarrhea compared to standard infant formula (10 vs. 19, *p* = 0.06), less episodes of diarrhea (49 vs. 97, *p* = 0.001). Respiratory tract infection was similar between groups (36 vs. 37).

AEs: Adverse events, FERM: Fermented formula, FOS: Fructo-oligosaccharides, GA: Gestational age, GOS: Galacto-oligosaccharides, LAB: Lactic acid bacteria, ORS: Oral rehydration solution, SD: Standard deviation, QOL: Quality of life.

**Table 2:** Studies with postbiotics later in life, including adults and elderly (>18 years).

Study	Type of study	Intervention	Comparison	*N* (age)	Population	Duration	Outcome
Tapiovaara et al. 2016 [99]	Double-blind, placebo controlled randomized, pilot study	Juice containing live or heat inactivated *L. rhamnosus* GG	Control juice without live or heat inactivated bacteria	59 (18–65 years)	Healthy subjects	6 weeks	A tendency towards the lowest HRV loads in the live *L. rhamnosus* GG group, and highest in the placebo group (on day 2: Live 6.20, heat inactivated 6.30 and placebo 7.25, *p* = 0.57). HRV load positively correlated with symptom scores (*p* = 0.034)
Sawada et al. 2016 [93]	Double-blind placebo controlled randomized study	Fermented milk beverage with sterilized *L. gasseri* CP2305	Artificially acidified milk-based placebo beverage	39 (20–70 years of age)	Healthy individuals with a tendency towards constipation (*N* = 20) or frequent bowel movements (*N* = 19)	3 weeks	In the intervention group, scores on the Bristol stool scale improved significantly after 3 weeks of intervention (*p* < 0.05). Output and color tone were also improved, especially in subjects with a tendency towards constipation. Moreover, SCFAs (propionate, butyrate and valeric acid) were significantly increased as well as *Clostridium* cluster IV and a beneficial effect on the regulation of intestinal function was found.
Shinkai et al. 2013 [97]	Double-blind placebo controlled randomized study	Tablets containing heat killed *L. pentosus* b240 in a low (2 × 10^9^) or high (2 × 10^10^) dose	Placebo tablets without *L. pentosus* b240	280 (>65 years)	Healthy elderly	20 weeks	Results for high dose vs. low dose vs. placebo were, for the accumulated incidence rate of common cold: 29.0% vs. 34.8% vs. 47.3% respectively (*p* for trend = 0.012). General health perception, measured by SF-36, increased in both intervention groups (*p* for trend = 0.016).
Arimori et al. 2012 [98]	Randomized single-blind placebo-controlled study	Tablets containing heat-killed *L. plantarum* L-137	Placebo tablets without *L. plantarum* L-137	16 (45.4 ± 8.1 years)	Healthy women	8 weeks	No differences were found between groups in seroresponse rate and geometric mean Ab titers after the first or second dose of inactivated influenza vaccine. Levels of IFN-β were significantly higher in the intervention group than in the placebo group (*p* < 0.05). Moreover, type I IFN production was enhanced in the intervention group.
Terrerias et al. 2011 [94]	Observational study	Inactivated *Lactobacillus* LB and fermented culture medium (Lacteol LB)	n.a.	297 (53.4 ± 17.3 years)	Patients with IBS-D	1 month	Pain scores decreased from 4.46 ± 0.15 to 2.8 ± 0.14 after treatment (*p* < 0.0001), as well as bloating from 4.49 ± 0.18 to 2.5 ± 0.15 (*p* < 0.0001). The HRQOL score, inversely correlated with quality of life, decreased from 5.99 ± 0.14 to 3.92 ± 0.16 (*p* < 0.0001). Mean number of stools per week also decreased from 17.59 to 12.83 after treatment (*p* < 0.0001).
Moroi et al. 2011 [96]	Double-blind placebo controlled randomized study	Heat-killed *L. paracasei* K71	Placebo without *L. paracasei* K71	34 (20–65 years)	Patients with mild or moderate atopic dermatitis	12 weeks	Skin severity scores decreased significantly in the intervention group, not in the placebo group (*p* < 0.05). Itch scores and QOL was not significantly different between groups. Consumption of topical therapeutics was 1.9 times higher compared to the intervention group, but not significantly different.
Xiao et al. 2002 [95]	Prospective, randomized trial	Live or heat-killed *L. acidophilus* LB	n.a.	137 (17–92 years)	Patients with chronic diarrhea	4 weeks	Mean bowel frequency was significantly lower in the heat-killed *L. acidophilus* LB group compared to the live *L. acidophilus* LB group (1.88 ± 1.24 vs. 2.64 ± 1.12 at week 2 and 1.39 ± 0.92 vs. 2.19 ± 1.05 at week 4, *p* < 0.05). Improvement in stool consistency, abdominal pain, distention and feeling of incomplete evacuation were significantly higher in the heat-killed *L. acidophilus* LB group.

Ab: Antibody, AEs: Adverse events, HRV: Human rhinovirus, HRQOL: Health related quality of life, IBS-D: Irritable bowel syndrome, diarrhea predominant, IFN-β: interferon- β, SCFAs: Short-chain fatty acids, SD: Standard deviation, QOL: Quality of life.

## Abbreviations

AEs	Adverse events
BHT	Breath hydrogen test
CID	common infectious disease
EPS	Extracellular polysaccharides
EVs	Extracellular vesicles
FAO-WHO	Food and Agriculture Organization of the United Nations—World Health Organization
FIFs	Fermented infant formulas
F6PK	fructose-6-phosphate phosphoketolase
GPRs	G-protein coupled receptors
GRAS	Generally recognized as safe
HDAC	Histone deacetylase
HePS	Heteropolysaccharides
HoPS	Homopolysaccharides
HMOs	Human milk oligosaccharides
HRV	Human rhinovirus
IBS	Irritable bowel syndrome
IFN	interferon
LAB	Lactic acid bacteria
lcFOS	Long-chain fructooligosaccharides
MVs	Membrane vesicles
OMVs	Outer membrane vesicles
ORS	oral rehydration solution
PEP	Phosphoenolpyruvate
PRQLQ	pediatric rhino conjunctivitis quality of life questionnaire
SCFAs	Short-chain fatty acids
scGOS	Shot-chain galactooligosaccharides
SIgA	secretory immunoglobulin A
TLR	Toll-like receptors
TNF-α	Tumor Necrosis Factor α
UC	Ulcerative colitis
